# Expanding thermoelectric horizons: Establishing correlations between thermoelectric parameters and multifunctional sensing signals

**DOI:** 10.1016/j.isci.2025.113549

**Published:** 2025-09-13

**Authors:** Bangzhou Tian, Ran Ang

**Affiliations:** 1 Key Laboratory of Radiation Physics and Technology, Ministry of Education, Institute of Nuclear Science and Technology, Sichuan University, Chengdu, Sichuan 610064, China; 2 College of Physics, Sichuan University, Chengdu 610064, China; 3 Institute of New Energy and Low-Carbon Technology, Sichuan University, Chengdu 610065, China

**Keywords:** Physics, Engineering, Materials science

## Abstract

Thermoelectric (TE) materials have traditionally been explored for power generation and solid-state cooling. However, issues such as toxicity, limited conversion efficiency, poor long-term stability, and high cost continue to hinder their large-scale deployment. Recently, the intrinsic capability of TE materials to convert thermal stimuli into electrical signals has garnered increasing attention for multifunctional sensing applications, including tactile feedback, respiration monitoring, and remote thermal detection. TE-based sensors offer distinct advantages, such as fast response, high durability, and excellent signal reproducibility. In this perspective, we highlight three strategically important research directions to advance TE sensing: (1) investigating carrier dynamics during TE transduction, (2) correlating TE material performance with key sensing metrics via compositional and defect engineering, and (3) integrating materials design with machine learning-assisted optimization. Advancing these frontiers will lay the groundwork for next-generation TE sensors in the Internet of Things, personalized healthcare, and intelligent industrial monitoring.

## Graphical abstract

**Figure:**
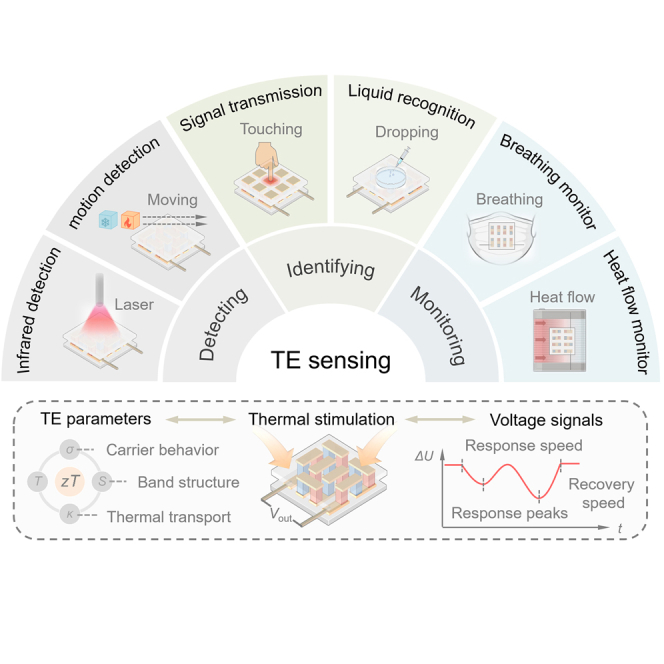

## Main text

Thermoelectric (TE) technologies, based on the Seebeck and Peltier effects, enable direct and reversible thermal-to-electrical energy conversion, offering versatile and sustainable solutions for energy applications.^1^^,^^2^ Current research predominantly focuses on optimizing TE materials and refining device designs for waste heat recovery and solid-state cooling applications.^3^^,^^4^ Central to these advancements is the pursuit of a high dimensionless figure-of-merit *zT*, defined as *zT* = *σS*^2^*T*/*κ*, where *σ*, *S*, *T*, and *κ* represent electrical conductivity, Seebeck coefficient, absolute temperature, and thermal conductivity, respectively.^5^ Achieving a high *zT* requires intricate balancing of these interdependent properties. Recent advancements in materials science and fabrication techniques have yielded notable breakthroughs. For example, a peak *zT* of ∼3.0 has been demonstrated in Cu_2_Se-based systems, alongside TE device conversion efficiencies approaching ∼13.4% under optimized conditions.^6^ Beyond Bi_2_Te_3_, significant cooling temperature differences exceeding 70 K at room temperature have been achieved using PbSe-based and Mg_3_(Sb, Bi)_2_-based materials.^7^^,^^8^ In the flexible TEs, a multi-heterojunction conjugated polymer exhibits an extraordinary *zT* value of 1.28 at 368 K,^9^ surpassing many commercial TE materials in performance. However, practical deployment faces persistent barriers, including the toxicity of Pb, the chemical reactivity of Mg (complicated process and low yields), the high cost of Ge, the stability of organic materials, and the mechanical limitations of Bi_2_Te_3_ (device fracture and failure). Moreover, moderate conversion efficiencies and inadequate long-term stability under operational conditions constrain commercial scalability.

**Figure 1:**
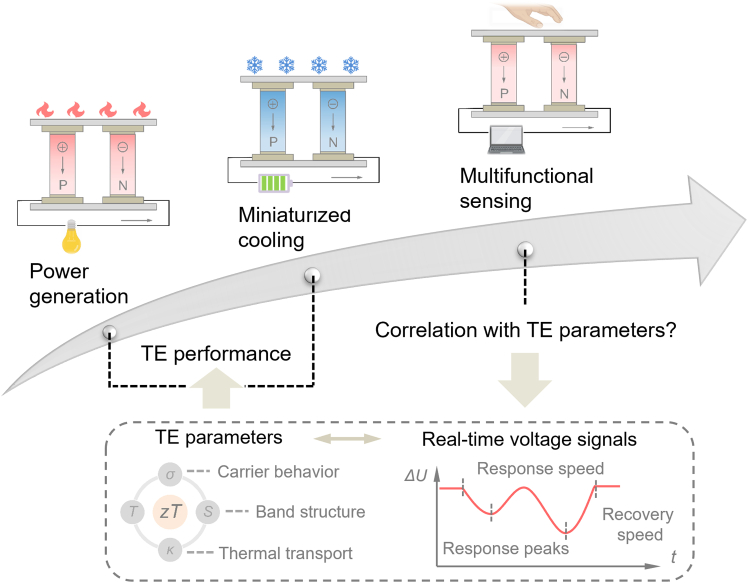
The evolution of thermoelectric technologies spans power generation, solid-state cooling, and real-time voltage sensing, unveiling intricate correlations between thermoelectric parameters and sensing signals

The fundamental voltage output of TE materials originates from carrier migration induced by temperature gradients, offering real-time responsiveness intrinsically linked to dynamic thermal environments.^10^ This inherent temperature sensitivity, combined with direct thermal-to-electrical signal conversion, presents unique opportunities for TE-based sensing technologies (Figure 1). Although insufficient for driving high-power devices, the strong correlation between TE voltage and thermal fields supports innovative applications in miniaturized, wearable sensing devices. The sensitive, rapid, and reproducible signal response achievable with low-voltage TE sensing reduces the demand for large material quantities. Also, TE sensing devices are typically not subjected to high-pressure environments, lowering the need for exceptional mechanical robustness. Recent research on Mg_3_(Sb, Bi)_2_-based materials highlights the rapid and repeatable open-circuit voltage responses to applied temperature differentials, demonstrating multifunctional sensing capabilities in diverse scenarios such as remote detection, tactile feedback, liquid recognition, and respiration monitoring.^11^

These advances herald a shift from traditional TE energy conversion toward interdisciplinary multifunctional applications. Solid-state TE sensors are emerging as promising candidates for real-world applications due to their circuit-free operation, rapid response times, stable signal outputs, and robust anti-interference characteristics. However, TE sensing research remains in its infancy, predominantly emphasizing bulk material characterization and reporting rudimentary device voltage signals.^12^ The underlying mechanisms governing how carrier dynamics dictate sensing signal generation, as well as the quantitative relationships between TE material parameters (e.g., *σ*, *S*, *κ*) and critical sensing metrics such as response speed, signal amplitude, and recovery rate, are poorly understood. Addressing these knowledge gaps is imperative for unlocking the full potential of TE sensing technologies.

To establish robust theoretical foundations and enhance TE sensing performance, the following research directions are proposed. (1) Unraveling carrier dynamics in voltage signal evolution. Investigating carrier migration states during voltage signal excitation, stabilization, and recovery is critical. Experiments with representative near-room temperature TE materials such as Bi_2_Te_3_ or Mg_3_ (Sb, Bi)_2_ under fixed infrared heat sources can record real-time voltage profiles while modulating temperature gradients via source distance or energy density adjustments. Designing or selecting suitable infrared heat monitoring systems enables accurate control of heat absorption in TE materials. Complementary density functional theory calculations and finite element simulations can provide insights into carrier behavior during these stages.^13^ (2) Quantifying correlations between TE parameters and sensing metrics. By systematically doping pristine TE materials to modulate individual TE parameters while preserving their band structures and/or microstructures, the dependencies of sensing metrics (e.g., voltage amplitude and response speed) on *σ*, *S*, and *κ* can be elucidated. Statistical analysis of controlled experiments will clarify whether these metrics are governed by single-parameter influences or synergistic interactions. Furthermore, adjusting carrier migration and thermal transport processes allows selective control of specific TE parameters, helping to reveal how individual TE properties influence sensing responses. (3) Optimizing sensing performance. Building on established correlations, strategies should focus on simultaneously maximizing response speed, signal amplitude, and recovery rate. Advanced optimization techniques, integrating machine learning and artificial intelligence algorithms,^14^ can address challenges across multiple scales—from microscopic carrier dynamics and material design to macroscopic composition engineering. In device design, utilizing thin films and casings with excellent thermal insulation reduces interference from ambient thermal fluctuations. Additionally, optimizing TE material geometry (size and shape) and wire-contact configurations according to application scenarios enhances signal acquisition accuracy.

**Figure 2:**
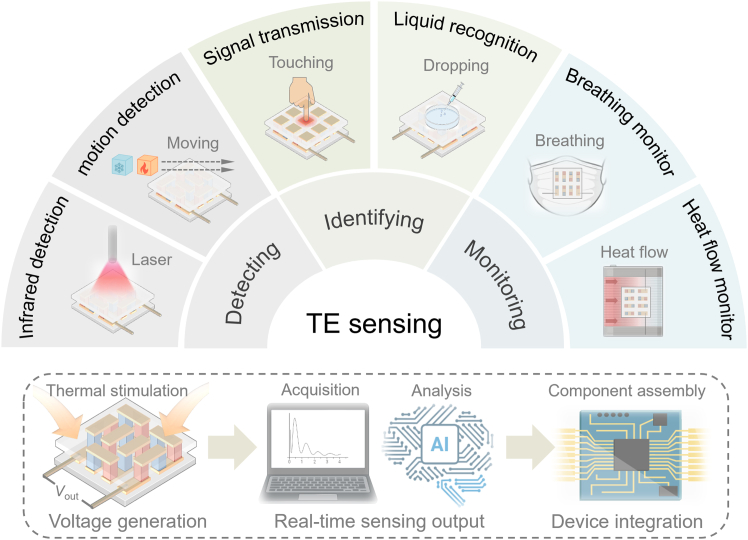
Multifunctional applications driven by thermoelectric sensing open new frontiers across diverse scenarios, emphasizing its transformative potential

Advancing TE sensing technology and developing robust modular devices open transformative possibilities for traditional TE materials in next-generation sensing applications. Figure 2 illustrates potential applications of future TE sensing. (1) Detecting. By leveraging the positive correlation between heat sources and TE signal intensity, TE sensors can detect infrared intensity (i.e., the power of infrared sources) and track thermal movements of objects, including direction and distance. (2) Identifying. TE sensing can achieve identification through specially designed sensor structures that encode distinctive signal responses. For example, intrinsic thermal parameters of different liquids allow differentiation and recognition of liquid types. (3) Monitoring. Heat-flow-sensitive TE sensors can perform continuous monitoring of dynamic processes. Representative applications include assessing respiratory status (where characteristic signal patterns correspond to different breathing behaviors) and equipment temperature monitoring (where abrupt signal changes indicate abnormal thermal conditions).

The rapid growth of the Internet of Thing, intelligent health monitoring, and wearable devices underscores the need for miniaturized, low-power, and highly stable sensors. Concurrently, TE sensors can play pivotal roles in industrial equipment monitoring, fire warning systems, and sewage treatment, providing innovative support for energy conservation and environmental sustainability.

By addressing the existing challenges and fostering interdisciplinary collaboration, TE technologies can evolve from energy conversion tools to multifunctional sensing solutions, transforming industries and enriching human life.

## Acknowledgments

The authors acknowledge support from the 10.13039/501100012166National Key Research and Development Program of China (grant no. 2022YFB3803900), the Guangxi Key Research and Development Program of China (grant No. GuiKe AB25069373), the Regional Innovation Cooperation Project of the Sichuan Science and Technology Program (grant no. 2024YFHZ0204), and the Sichuan University Innovation Research Program of China (grant no. 2020SCUNL112).

## Author contributions

B.T., investigation, formal analysis, visualization, and writing – original draft; R.A., conceptualization, formal analysis, writing – review and editing, supervision, and project administration.

## Declaration of interests

The authors declare no competing interests.
